# 

**Published:** 1897-10

**Authors:** 


### CONTENTS:

### SELECTIONS.

Article I.—The Clinic. By E. P. Beadles, D. D. S.... 241
" II.—To What Extent and When are We Justified in Using Cataphoresis? Is There Danger of Injuring the Dental Pulp or Other Tissues by its Use? By H. T. Harvey, D. D. S.... 243
" III.—Union of the American and the Southern Dental Associations.... 253
" IV.—Treatment of Pulpless Teeth. By A. J. Husband, L. D. S.... 260
" V.—Resection and Reproduction of the Maxillæ. By G. Lennox Curtis, M. D.... 263
" VI.—Point of Contact. By R. W. Morse, D. D. S.... 272
" VII.—Cataphoresis. By Dr. C. L. Hungerford.... 275

### COMMUNICATED.

Northern Illinois Dental Society.... 283

### MONTHLY SUMMARY.

Acetanilide as a Dressing.... 284

A Simple Way of Putting on Metal Backings for Vulcanite Work Without Soldering.... 285

A Good Law.... 285

Discussion on Local Anesthesia.... 286

Kaiserling's Method for the Preservation of Specimens with their Natural Colors.... 287

Medical Superstitions.... 287

A New Hemostatic.... 287

To Open Pulp Chambers of Teeth Affected with Pericementitis.... 288

A Hint on Extracting.... 288

Ammonol.... 288

